# Features of the Synthesis of the Dispersed TiC Phase with Nickel Nanostructures on the Surface to Create an Aluminum-Based Metal Composite

**DOI:** 10.3390/nano11102499

**Published:** 2021-09-25

**Authors:** Elena G. Zemtsova, Denis V. Yurchuk, Pavel E. Morozov, Petr M. Korusenko, Vladimir K. Kudymov, Vladimir M. Smirnov

**Affiliations:** Institute of Chemistry, Saint Petersburg State University, 7/9 Universitetskaya nab., 199034 Saint Petersburg, Russia; 667_766_d@mail.ru (D.V.Y.); comitcont@yandex.ru (P.E.M.); korusenko_petr@mail.ru (P.M.K.); v.k.kudymov@gmail.com (V.K.K.); vms11@yandex.ru (V.M.S.)

**Keywords:** titanium carbide, nickel nanostructures, nanoparticles, electroless deposition, aluminum matrix composites

## Abstract

The development of new composites with improved functional properties is the important task of modern materials science. The composites must be structurally organized to provide improved properties. For metal-ceramic composites, there is a need for a uniform distribution of the dispersed ceramic phase in the bulk metallic matrix The modification of the dispersed ceramic phase surface with a metal coating is one of the more effective ways to accomplish this. Particularly, in this work, the conditions of Ni nanolayer deposition on titanium carbide (TiC) particles were studied. The goal was to create core–shell particles with a thickness of the Ni coating on TiC not exceeding 90 nm. Preliminary work was also carried out to study the effect of the dispersed phase composition on the mechanical properties of the composite with an Al matrix.

## 1. Introduction

Aluminum alloys and steels have remained the basic structural materials for mechanical engineering and modern aviation equipment [1,2,3,4]. At the same time, there is a need to create new heat-resistant metal composites, including those based on 3D-structuring of a metal matrix by carbide nanostructures. This is dictated by increasing requirements for their properties. Metal matrix composites (MMCs) have attracted a lot of attention in recent years due to their improved mechanical properties, such as their specific strength [4,5]. In particular, composites consisting of nanoparticles as the second phase dispersed in the matrix grains and at the grain boundaries [6], have now become objects of interest due to their improved mechanical, chemical, and physical properties. However, traditional consolidation methods, such as high-temperature sintering and hot pressing, have significant limitations, e.g., the inability to hold nanoscale grains due to excessive grain growth during high-temperature processing [7].

At the moment, a number of research groups develop new MMCs obtained by casting with mixing to avoid sedimentation and aggregation of the reinforcing component [8,9,10,11]. Furthermore, development continues of both liquid-phase methods and methods of powder metallurgy [12,13].

The promising directions for obtaining workpieces for mechanical engineering from composites are liquid-phase and powder metallurgic methods. The advantages of these methods are the shortened production cycle and simpler standard equipment. Moreover, it becomes possible to use not only clean components but also secondary raw materials.

However, the uneven distribution of particles over the bulk composite is undesirable here. This is due to the fact that composites reinforced with a dispersed ceramic phase have poor wettability with liquid metal, which leads to agglomeration of ceramic particles and uneven particle distribution over the bulk composite.

One of the effective solutions of this problem is the modification of the dispersed ceramic phase surface [1,14].

Electroless metals deposition is a widespread method of obtaining metal coatings on various surface types [15,16]. So, this method was chosen for the metallic Ni deposition on the dispersed ceramic phase surface. For electroless deposition of metals, silicon carbide (SiC) powder is often used as a dispersed ceramic phase [17,18]. In this work, for the first time, titanium carbide (TiC) powder was used instead.

Our purpose was to study the conditions of a Ni nanolayer deposition on TiC, and the possibility to control its thickness. Also, the goal was to create core-shell particles, with the Ni/TiC coating thickness ≤ 90 nm. The effect of the dispersed phase composition on the mechanical properties of the Ni/TiC composite with Al matrix was also studied.

## 2. Materials and Methods

### 2.1. Materials

The following reagents were used in the work: tin(II) chloride dihydrate SnCl_2_·2H_2_O (Sigma-Aldrich, No. 243523, St. Louis, MI, USA), palladium(II) chloride PdCl_2_ (Sigma-Aldrich, No. 205885), concentrated hydrochloric acid HCl conc. (Sigma-Aldrich, No. 320331), nickel(II) chloride hexahydrate (Sigma-Aldrich, No.31462), sodium citrate dihydrate (Sigma-Aldrich, No. W302600), ammonium chloride (Sigma-Aldrich, No. 213330), sodium hypophosphite monohydrate NaH_2_PO_2_·H_2_O (Sigma-Aldrich, No. S5012), concentrated ammonia solution (Sigma-Aldrich, No. 1.05428).

Titanium carbide (TiC) powder (Sigma-Aldrich, No. 594849) was used as a substrate to deposit Ni nanolayers with an average particle size of ca. 0.8–1.5 micron. Al powder ASP-50 (United Company RUSAL, Volgograd, Russian Federation) with an average particle size of 50 microns was used as a matrix.

### 2.2. Mechanism of Formation of Ni Nanocoating on the TiC Particles Surface (TiC/Ni)

To obtain a Ni nanocoating on TiC particles, the TiC surface was activated using SnCl_2_ and PdCl_2_ solutions. This process is critical for the deposition of metallic Ni on the ceramic particles surface. Treatment of TiC particles with SnCl_2_ solution stimulates the adsorption of Pd^2+^ ions at the next step of the activation process. Sn^2+^ ions are adsorbed on the TiC surface, becoming nucleation centers for Pd during activation [19,20,21], namely, the Sn^2+^ ion reacts with the Pd^2+^ ion, leading to the formation of Pd catalytic centers on the surface. This reaction can be described by Equation (1):

Pd^2+^ + Sn^2+^ → Sn^4+^ + Pd^0^ (1)

The process of TiC particles activation occurred as follows: TiC powder was mixed with 50 mL of acetone, followed by centrifugation and the release of a precipitate from TiC, which was then dried in a drying cabinet at a temperature of 105 °C for 1–2 h. Next, 50 mL of SnCl_2_ solution was added to the dried TiC, followed by stirring to a suspension state for 10 min and centrifugation. Then the supernatant solution separated from the TiC. The 50 mL of PdCl_2_ solution was added to the resulting TiC precipitate and shaken to a suspension state, centrifuged for 10 min and then the supernatant solution was separated from the TiC. 

The reduced palladium atoms are remained on the TiC surface after the activation process. When the activated particles are introduced into the NiCl_2_ solution, an autocatalytic reaction begins when NaH_2_PO_2_ is added, which can be represented as Equation (2):

Ni^2+^ + H_2_PO_2_^−^ + H_2_O → H_2_PO_3_^−^ + H_2_↑ + Ni^0^↓ (2)

Equation (2) shows that Ni^2+^ ions are reduced to metal. Nanoscale Ni particles are formed in the near-surface layer of TiC particles and tend to aggregate. The reduced Ni particles are electropositive due to the sorption of Ni^2+^ ions from the solution. A reaction between Pd nuclei on the TiC surface and Ni^2+^ ions on the surface of Ni nanoparticles (NPs) is also possible, which can be described by Equation (3):

Pd^0^ + Ni^2+^ → Pd^2+^ + Ni^0^ (3)

In this reaction, Ni is also reduced by Pd and attached to the surface of the original TiC, forming a Ni layer on the TiC surface (TiC/Ni-1 sample). Ni coatings were also obtained after 3 cycles of Ni deposition on TiC particles (TiC/Ni-3 sample). The composition of the solutions and the deposition conditions are given in Table 1.

### 2.3. Powder Compaction

Preliminary, before using Al as matrices for the composite, Al powder was standardized in size, the metal surface was cleaned from oiling agents.

1. Aluminum ASP-50 was pre-sieved on a vibrating screen VP-30T using a sieve with a cell size of 80 microns. A larger fraction was rejected.
2. A suspension of Al (100 g) in 500 mL of isopropyl alcohol was shaken, filtered, dried in air, finally dried under vacuum at room temperature.

Composites were obtained by powder metallurgy. Aluminum powder ASP-50 was mixed with 5 wt.% TiC/Ni or TiC particles (Al + 5% TiC and Al + 5% TiC/Ni samples). The components were mixed in a planetary ball mill PM 100 CM using steel balls with a diameter of 5 cm. The powder: balls ratio was 1:25. Also, as the blank specimen, the material containing ASP-50 aluminum only, was prepared.

Sample (1.75 g) was compacted using a Mega PRS15 hydraulic manual press, under a load of 3.5 GPa at room temperature. The sample was kept under load for 10 min. The thickness of the disk is 1.95–2.05 mm.

Both the blank and composite disks were compacted by cold pressing on the Walter Klement GmbH HPT-07 press. For pressing, strikers with a sample depth of 0.6 mm and a sample diameter of 20 mm were used. Pressing was carried out at room temperature under a pressure of 6 GPa, the workpiece was kept under pressure for 15 min. The thickness of the disk was ~1.8 mm. The pressed disk was placed in a tubular quartz reactor, kept at 200 °C for 2 h, after which the temperature was raised to 600 °C and kept for another 2 h. Sintering was carried out in a hydrogen atmosphere.

### 2.4. Characterization

Morphology and elemental analysis were performed using a Zeiss Merlin scanning electron microscope (SEM) with additional setups for X-ray microanalysis (Carl Zeiss AG, Jena, Germany). The elemental analysis was taken for dry particles deposited on a single-crystal Si substrate.

X-ray diffraction (XRD) analysis was performed on a Bruker D2 Phaser powder diffractometer (Bruker Corporation, Billerica, MA, USA). The phases in the samples were identified using the data of the International Center for Diffraction Data.

Rapid qualitative analysis of the elemental composition was performed on an energy-dispersive X-ray fluorescence spectrometer of the EDX 800 HS series (Shimadzu, Kyoto, Japan).

To estimate the mass of the formed coating, samples were weighed (in a dry state) before and after synthesis, as well as before and after calcination. The results of the mass change during synthesis are presented in Table 2.

### 2.5. Mechanical Tests, Tests for Uniaxial Tension

Double-sided blades with a working part size of 6 × 2 mm were made from prepared samples using the ART 123 PRO electroerosion machine (“Delta-Test” LLC, Fryazino, Russia).

The blade for mechanical tests has a flat double-sided shape with a thickness of not more than 1.8 mm.

The width of the sample heads is 6 mm, the height of the working part of the head that provides grip with the clamps of the test machine is 2 mm.

The ends of the blades were examined using a Micmed-6 microscope with side illumination in order to control the samples integrity.

Hydrostatic weighing on Shimadzu AUW220 scales with the SMK-401 density determination kit was used to determine the densities.

Uniaxial stretching was carried out on a Shimadzu AG-50kNX testing machine at room temperature, the deformation rate was 5·10^−4^ s^−1^. The samples deformation was measured by a video extensometer TRViewX 55S ("Shimadzu Corporation", Kyoto, Japan). Uniaxial tension was measured in accordance with the procedure described in Russian state standard GOST 1497-84 “Metals. Methods of tension test”.

## 3. Results and Discussions

### 3.1. SEM and EDX

The Ni surface structures on TiC were studied by EDX and SEM. EDX was used for rapid assessment of the presence/absence of Ni in the composition after synthesis. In Table 3, data for the initial TiC particles before and after 1st Ni precipitation cycle (TiC/Ni-1 sample) are given. According to EDX (Table 3), initial TiC contains Fe admixtures. After synthesis, considerable amounts of Ni and phosphorus-containing impurities (from the reaction mixture) and Pd and Sn (adsorbed during activation) were found.

**Figure 1:**
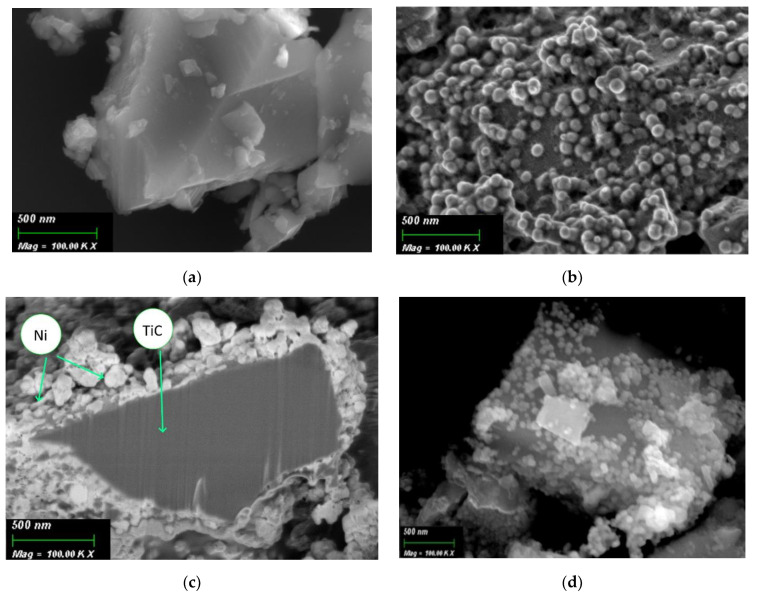
SEM images: (**a**) initial TiC; (**b**) TiC/Ni-1 sample; (**c**) cross-section of a TiC particle with Ni NPs on the surface; and (**d**) TiC/Ni-3 sample.

Figure 1a shows a micrograph of the initial TiC particle. As can be seen in Figure 1a, TiC is a large crystalline particle of micron sizes (20–50 microns) with a flat surface. In TiC/Ni-1 sample, the TiC surface changes. From Figure 1b, it can observe a rough surface layer consisting of globules of the order of several tens of nanometers in size (50–70 nm). Figure 1b shows that a surface layer of spherical Ni NPs has formed on the TiC particle surface. Figure 1c shows cross-section of TiC particle with Ni NPs on the surface. Thanks to this cross-section, it is possible to estimate the thickness of the surface layer of spherical Ni NPs (70–150 nm). In TiC/Ni-3 sample, the deposited Ni NPs tend to agglomeration. As can be seen from Figure 1d, the TiC particle surface is covered with pronounced spherical Ni particles that form agglomerates with a size of ca. 150–200 nm.

**Figure 2:**
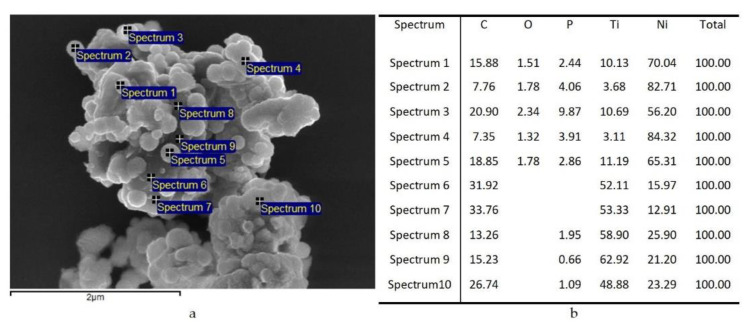
(**a**) SEM image of a TiC particle surface with Ni structures; (**b**) data obtained from elemental analysis spectra recorded at the corresponding points on the TiC/Ni particle (TiC/Ni-1 sample).

For a detailed study of the TiC particles surface with Ni nanostructures, the elemental composition of samples at several surface points was studied with microscopy (Figure 2). On the TiC surface micrograph, we observe a uniform distribution of Ni NPs. 

An average assessment of the elemental composition was carried out for initial TiC, TiC/Ni-1, and TiC/Ni-3 samples, the composition data are given in Table 4.

As can be seen from Table 3, after one deposition cycle, the element map of the sample surface changes. According to Table 4, the average Ni content on TiC/Ni-1 surface is ca. 14 at.%. Also, the composition shows the appearance of ca. 6 at.% P, which could be captured from the solution as various Na and Ni phosphates. Interestingly, on TiC/Ni-3 surface, the Ni content remains almost the same as in TiC/Ni-1 sample, but the deposited Ni particles undergoes agglomeration (Figure 1d).

SEM does not allow to unambiguously assess in what form Ni presents in the composition—in the form of a metal or in other forms. Therefore, XRD was further carried out to clarify this issue.

### 3.2. XRD Analysis

**Figure 3:**
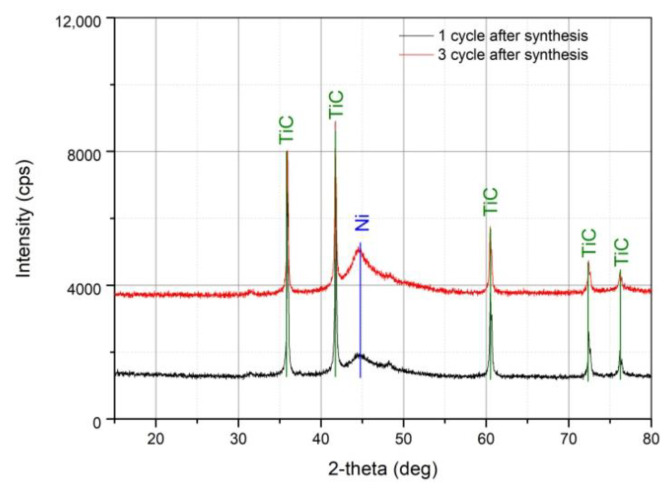
XRD patterns of TiC/Ni-1 and TiC/Ni-3 samples.

Samples before and after calcination were analyzed by XRD (Figure 3). Typical TiC peaks (JCPDS card 01-071-0298, Khamrabaevite) were detected in the pattern of non-calcined TiC/Ni-1 and TiC/Ni-3 samples (Figure 3) at 35.9567, 41.7533, 60.506, 72.416, and 76.197°. A fairly wide peak is observed at ca. 45°, which is attributed to the characteristic Ni (111) peak (JCPDS card 01-071-4654, Nickel) at 44.48°.

The absence of a distinct Ni peak suggests that the as-synthesized material is amorphous. But even in this case, there is a tendency for the Ni peak to increase with an increase in the cycles number.

Ni crystallite size in the samples before calcination was roughly evaluated using Debye-Scherrer equation. The Ni crystallites in TiC/Ni-1 and TiC/Ni-3 samples before calcination are close in size (92 and 102 Å, respectively). The area of Ni peak at ca. 45° for TiC/Ni-3 is ca. 1.5 times higher of that for TiC/Ni-1. So, Ni peak intensity and area indicate increase of Ni content in the TiC/Ni-3 sample rather than increase of Ni crystallite size. This is also confirmed by the increase of Ni/Ti ratio with increasing of cycles number.

Angles characteristic of the components of the reaction mixture NiCl_2_ (JCPDS card 01-071-2032), reaction products Ni_3_(PO_4_)_2_ (JCPDS card 01-074-8719), Ni(H_2_PO_2_)_2_(H_2_O)_6_ (JCPDS card 01-075-0030) and oxidation products after synthesis—rutile (JCPDS card 01-073-1232), anatase (JCPDS card 01-071-1166), NiO (JCPDS card 01-071-1179) were not found.

**Figure 4:**
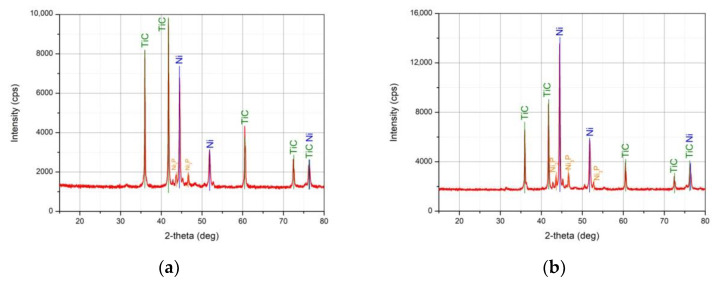
XRD patterns of (**a**) TiC/Ni-1 and (**b**) TiC/Ni-3 samples after calcination at 500 °C under H_2_ stream.

To crystallize nickel, the samples were calcined at 500 °C for 1 h in a H_2_ stream. XRD patterns of the calcined samples are shown in Figure 4. Distinct Ni peaks are observed at angles of 44.48, 51.83 and 76.35°. At 76.35°, the Ni peak is superimposed with the characteristic TiC peak at 76.197°. It is necessary to note the peaks of Ni_3_P impurity, which could be formed during the hydrogen reduction of phosphorus-containing salts that were not pre-washed. This indicates the need for more thorough washing of synthesized samples.

The Ni crystallite size for samples after calcination was evaluated using the same method as for non-calcined ones before. For TiC/Ni-1 and TiC/Ni-3 crystallite size is about 342 and 364 Å, respectively. Thus, calcination leads to crystallite size increase in several times compared with non-calcined samples while the peak areas ratio remains almost the same for calcined and non-calcined samples.

Phase ratio for Ni and TiC is different for TiC/Ni-3 and TiC/Ni-1 while Ni phase ratio for TiC/Ni-3 and TiC/Ni-1 samples before and after calcination are 2 and 1.98, respectively.

When superimposing XRD patterns of calcined samples, it is seen that Ni crystallizes and clear Ni peaks are traced both for TiC/Ni-1 and TiC/Ni-3 samples. It is also evident that the Ni content in TiC/Ni-3 sample is noticeably higher, and the Ni_3_P impurity amount is also higher. The formation of closed pores with impurities in the Ni shell leads to a significant increase in the sample mass (101.3%, Table 2). In order to avoid uncontrolled blockage of Ni_3_P impurity, further we used TiC/Ni-1 samples only.

Two XRD peaks at ca. 32 and 48° were not identified. After calcination these peaks maintain the same intensity as compared to non-calcined samples (Figure 3 and Figure 4). 

### 3.3. Mechanical Uniaxial Stretching Tests

To study the effect of the TiC/Ni nanostructures on the mechanical properties of the Al matrix composite, TiC/Ni-1 samples were selected. This approach shows a uniform distribution of Ni NPs on the TiC surface. The mechanical properties of aluminum-matrix composites were studied by uniaxial stretching. For comparative studies, samples were also made of pure dispersed Al and one of a metal matrix material containing TiC particles without Ni shell.

The powder material was compacted and sintered in the form of a blank disk with a residual internal porosity of 98%.

**Figure 5:**
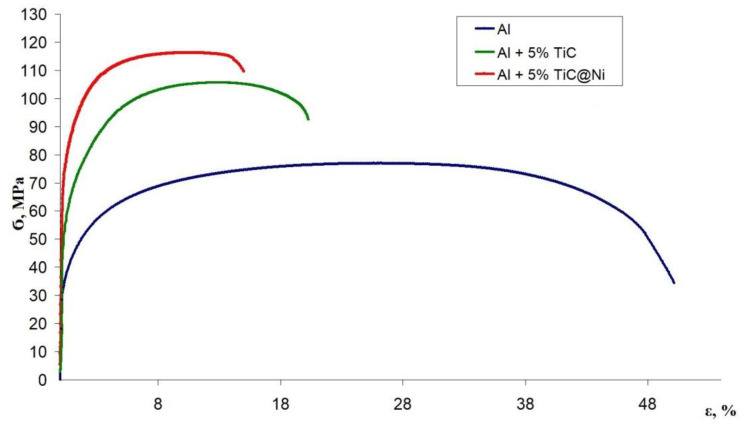
Stretching diagrams of aluminum matrix composite samples of the compositions: Al, Al + 5% TiC, Al + 5% TiC/Ni.

Al + 5% TiC and Al + 5% TiC/Ni samples were compared (Figure 5). The data obtained during the mathematical processing of the stretching diagrams for the studied samples are shown in Table 5.

The Al + 5% TiC composite demonstrates a significant increase of the ultimate strength (106 MPa) related to the pure Al (76 MPa). This is explained by the TiC influence on the structure and properties of the Al matrix. TiC particles interact with the grain boundaries of the Al matrix and form so-called hardening zones around themselves, since they are stress concentrators, thereby preventing the material destruction. However, TiC dispersed phase has a significant disadvantage. At high concentrations of dispersed particles (>3 vol.%) there is an undesired aggregation in the composite and the impossibility of uniform distribution in the Al matrix. These aggregates provoke the appearance of unnecessary defects and pores, which limits the possibility of increasing the strength properties of such composites.

For Al + 5% TiC/Ni composite we observe a significant increase in the tensile strength (116 MPa) compared to pure Al (76 MPa) and to Ni-free composite Al + 5% TiC (106 MPa). This composite advantage is explained by the better TiC/Ni particles distribution in the Al matrix, compared to Ni-free TiC particles. At the same time, the increase in the tensile strength could be affected by the formation of local Guinier–Preston zones at the “TiC/Ni–Al matrix” boundary, due to the penetration of Ni atoms into the Al matrix during the sample sintering. The radius of Ni and Al atoms are close (Ni-0.124 nm, Al-0.143 nm). In this case, the direct influence of the carbide core of the ligature on the composite matrix is significantly reduced, due to the shielding with a Ni shell, which is confirmed by a slight decrease in the yield strength of this material.

It is also worth noting that the mechanical properties of aluminum matrix materials obtained from spherical aluminum are also most likely associated with dispersion hardening with aluminum oxide by the Orovana mechanism [22,23,24]. At the same time, the absence of excessive growth of deformation hardening of the material is consistent with the work [25].

Thus, in the course of the study, the application of electroless metals deposition for the production of core-shell particles was demonstrated, where titanium carbide (TiC) powder was used as a dispersed ceramic phase. Ni peak intensity and area indicate increase of Ni percentage after 3 deposition cycles rather than increase of Ni crystallite size. This is also confirmed by the increase of Ni/Ti ratio with the increasing of the cycles number. Furthermore, this confirms an increase in the degree of Ni crystallinity.

Our experimental results demonstrate that the chosen method for production of the particular composite with 5% TiC/Ni dispersed phase allows the obtaining of other materials with improved mechanical properties comparable to industrial samples.

## 4. Conclusions

As a result of this work, the following new results were obtained.

Metallic nickel was synthesized on the TiC powder surface by electroless chemical deposition. After single synthesis cycle (with Pd activation), spherical Ni nanoparticles were obtained with a thickness of ca. 50–70 nm (SEM data). The thickness of the surface Ni layer consisting of spherical Ni NPs is 70–150 nm. XRD confirms that the nanostructures on the TiC surface contain amorphous Ni phase; no foreign salts and nickel oxides were detected. In this case, the crystalline Ni is formed only after post-processing of dispersed samples at 500 °C for 1 h in a H_2_ stream. It was also shown that an Al matrix composite containing 5 wt.% of the dispersed TiC phase with a Ni shell shows a significantly (1.5-fold) higher tensile strength. This behavior of the aluminum matrix composite is explained by the homogeneous composite microstructure due to a more uniform distribution of TiC/Ni particles in the matrix, compared with TiC particles. 

## Figures and Tables

**Table 1:** Compositions of the solutions used.

Solution	Component	Concentration
SnCl_2_ solution	SnCl_2_	10 g/L
	HCl conc.	30 mL/L
PdCl_2_ solution	PdCl_2_	0.25 g/L
	HCl conc.	3 mL/L
NiCl_2_ solution	NiCl_2_	45 g/L
	NH_4_Cl	50 g/L
	Na citrate	100 g/L
	NH_3_ conc.	until pH 8.5
	TiC	5 g/L

**Table 2:** Changes in the mass of the TiC/Ni sample during synthesis.

Cycles Number	Sample Code	TiC Weight	The Mass of the Sample after Drying	Weight Gain, %
1	TiC/Ni-1	1.96 g	3.23 g	64.8%
3	TiC/Ni-3	1.90 g	3.82 g	101.3%

**Table 3:** EDX qualitative analysis of TiC particles before and after 1st Ni precipitation cycle.

	Average Content			
Element	Intensity for Initial TiC	Average Content for Initial TiC, at.%	Intensity for TiC/Ni-1	Average Content for TiC/Ni-1, at.%
Ti	213.14	94.73	175.59	68.45
W	6.70	3.27	4.22	1.64
Ni	-	-	64.25	25.05
Fe	1.17	0.53	0.14	0.05
Co	1.66	0.77	-	-
Nb	0.66	0.32	0.57	0.22
Sn	-	-	7.26	2.83
Pd	-	-	2.93	1.64
P	-	-	1.10	0.43
Others	0.55	0.24	**1.03**	0.40

**Table 4:** Elemental analysis averaged over a series of TiC particle surfaces (initial one, TiC/Ni-1 and TiC/Ni-3 samples) determined by energy dispersive spectroscopy.

	Average Content, at.%		
Element	Initial TiC	TiC/Ni-1	TiC/Ni-3
Ti	52.02	32.70	49.17
P	-	5.67	-
Ni	-	13.65	13.30

**Table 5:** Summary of characteristics of aluminum matrix materials.

Number	Sample Composition	Ultimate Strength (σ), MPa	Density Ratio (exp/calc), %	Yield Strength, MPa	Relative Elongation (S), %
1	Al	76 ± 2.0	98.35	31.2 ± 0.2	26.20
2	Al + 5% TiC	106 ± 2.0	98.47	45.0 ± 0.2	13.20
3	Al + 5% TiC/Ni	116 ± 2.0	98.13	38.0 ± 0.2	7.50

## Data Availability

The main data had been provided in the paper and supplementary material. Any other raw/processed data required to reproduce the findings of this study are available from the corresponding author upon request.
